# Impact of button position and touchscreen font size on healthcare device operation by older adults

**DOI:** 10.1016/j.heliyon.2020.e04147

**Published:** 2020-06-30

**Authors:** Po-Chan Yeh

**Affiliations:** Institute of Creative Design and Management, National Taipei University of Business, No. 100, Sec. 1, Fulong Rd., Pingzhen Dist., Taoyuan City, 324, Taiwan

**Keywords:** Neuroscience, Cognitive aspect of human-computer system, Interaction design, Human machine interaction, User interface, Aging and life course, Older adults, Interface design, Ergonomics, Touchscreen, Healthcare device

## Abstract

In 1993, Taiwan officially became an aging society. Degradation of physiological function during aging is inevitable; visual and physical reactions are especially vulnerable. Given the popularity of electronic devices and their vigorous development in recent years, touchscreen use is now commonplace. As society ages, many people use devices to monitor their health. Some products have gradually eschewed their traditional interfaces, which have been replaced by touchscreens. Touchscreen operation and interpretation differ between older and younger populations. Here, we focus on healthcare equipment, exploring the effects of button position and touchscreen font size on operation by older people. To understand differences between older and younger people, we invited 32 people aged 18–35 years, and 32 people aged over 65 years, to participate in our experiment. Each subject tested four button positions and four font sizes, thus 16 test interfaces in all. We found that young people found no differences among the 16 interfaces, but the older group did. Larger fonts reduced operation time for older participants. At a font size of 22 pt, the older group performed as well as the young participants. When buttons were positioned at the top of the interface, the performance of the older participants improved. Overall, use of a font size of 22 pt and top-positioned buttons optimized the performance of the older participants while use of a font size of 10 pt and bottom-positioned buttons maximally degraded their performance. Our results can be used to design interfaces appropriate for older people, thus improving their autonomy.

## 1 Introduction

With improvements in nutrition and medical technology, the average human lifespan has increased significantly (Huang, 2004). According to the Ministry of Internal Affairs, Taiwan officially became an aging society in September 1993. This demographic phenomenon is evident in both developed and developing countries. In 2009, the average number of the older population was close to that of other major countries (Directorate-General of Budget, Accounting and Statistics, Executive Yuan, 2015). By 2030, the proportion of the population over the age of 65 years will be equal to those of Europe and North America, at 12–24%.

Technological progress has changed many aspects of life for the better (Jacelon and Hanson, 2013). Interface communication is now routine (Lee et al., 2015), affecting all aspects of society (Petrovčič et al., 2015; Moisescu, 2014; Im and Park, 2014; Rodrigues et al., 2014; Ryu et al., 2009). According to Czaja’s (2019) research, Information and Communication Technologies (ICT) can improve the quality of life and independence of the older population, especially considering that 42% of them own smart phones and 32% own tablet-type computers. There are also significant differences in the use of ICT by older people. The main reasons for low ICT use include cost, lack of confidence in the ability to learn the technology, physical challenges, and a reliance on others for training and technical support. The last factor, reliance on others, is the most common problem for older populations who live independently, have limited mobility, or live in rural areas (Anderson and Perrin, 2017).

If technology products are comfortable to operate and perform sufficiently well to increase quality of life (Czaja et al., 2006), their usage will increase. Aging compromises vigor and vitality (Paterson et al., 2007). Vision, hearing, physical capacity, and mental functioning decline with age (Pa et al., 2014; Goodpaster et al., 2006). In particular, visual sensory systems (Shrestha and Kaiti, 2014; Wang and Tsai, 2003; Lemme, 2002) and physical responsiveness becomes degraded over time (Goodpaster et al., 2006), slowing information processing and the appropriate behavioral responses.

In recent years, the touchscreen industry has grown tremendously, as it is characterized by intuitive and humanized operation rather than the use of traditional physical keys. Interactive operations differ from traditional interfaces; in currently available interaction models, older types (e.g., buttons, dials) are used concurrently with newer modes (e.g., voice, gesture), and coexist with touch-based interfaces. Touch is growing as the most prevalent and widely utilized mode of interaction. In this context, understanding the physical and mental characteristics of the older population will facilitate the design of products that facilitate their well-being.

## 2 Research background

The Ministry of Health and Welfare (2019) reports that 60.9% of Taiwanese aged 55–64 years suffer from chronic diseases, primarily high blood pressure, high blood lipids, osteoporosis, and diabetes, principally attributable to cardiovascular system deterioration (Angeli et al., 2014; Kapoor and Kapoor, 2013) that is evident in both developing and developed countries (Kearney et al., 2005). Therefore, in addition to increasing the frequency and duration of medical treatment, improvements in medical knowledge have caused self-health monitoring to grow in popularity. Because medical uses now constitute 20% of all home electronics (Institute for Information Industry & Multimedia Consumer Electronics Research Team, 2010), there is growing concern over the design of their interfaces, since proper operation is important for ease of use. Thus, understanding interface design is important for public health.

An electronic interface may feature text, images, and colors, rendering products attractive and improving interaction and usability. Optimal interfaces are reader-friendly (Yau et al., 2008), simple, and easy to operate (Lee et al., 2015; Choi and Lee, 2012). Elements affecting interpretation include message location and volume (Chen et al., 2003), and font size (Lee et al., 2011; Ziefle, 2010). Messages attract most attention when placed on the top middle and right, and may be ignored if placed on the bottom right. Bernard et al. (2003) compared 10- and 12-point fonts; the latter were preferred. Ramadan (2011) found that 14-point fonts on a white background enhanced readability.

As the Internet becomes universal, mobile phones and tablet computers have rendered interface communication a part of everyday life (Lee et al., 2015; Paulins et al., 2015; Wallace, 2012; Hein et al., 2011). Touchscreens are portable and can be operated anywhere (Billinghurst and Vu, 2015). The touch area is a major factor affecting operation (Huang and Lai, 2008), and has become a feature of concern to users (Jung and Im, 2015; Lindberg and Näsänen, 2003). Button number, size (Huang and Wu, 2015), and position (Huang and Lai, 2008) also affect operating performance (Lindberg et al., 2006). Lee et al. (2018), indicated that hand detection, hand-shape recognition, and hand tracking have been emerging topics in research on human-computer interaction (HCI).

As visual ability tends to decline with age, older users need more time to access information than young users (Oehl and Sutter, 2015; Wang et al., 2012). Presentation time and font size (Borg et al., 2015; Mahmud et al., 2010; Huang and Yeh, 2007) also have an impact on older users’ processing and interpretation of information. Also, for less experienced users (Kim et al., 2019), character display will affect frequent eye movements, which impacts information processing. Charness and Bosman (1990) found that older user groups preferred black and white characters and backgrounds over similar colored features, whereas younger user groups exhibited no such preference.

Unlike the physical tactile feedback given by traditional mechanical buttons in the past, touch-based interfaces are operated by users' fingers in more sophisticated actions, such as: press, tap, long press, and drag. Although more universal designs have slowly been introduced into typical interface designs, the interface interaction method still generates new usability problems. The most common issues for older populations stem from misinterpretation and misunderstanding. While research has examined use of buttons and other features on touch screens, most studies use young test subjects (Colle and Hiszem, 2004; O'Brien et al., 2008; Schedlbauer, 2007), or focus on mobile phones or tablet-type computers (Jin et al., 2007 Murata and Iwase, 2005). Research is needed to examine the implications of operational interfaces for other audiences and product types.

Many touchscreen products for patients (Jackson and Peters, 2003; Holzinger, 2003) and caregivers (Astell et al., 2010), and assistive devices, have enhanced patient care. As people age, many use home equipment to monitor their health. Some of these products no longer use traditional interfaces, opting for touchscreens. Differences in interpretation between older and younger people are inevitable. Many studies on the issues that older subjects have with touch interfaces have emerged, but most have focused on mobile devices or computer screens. The operation and interpretation of electronic medical products have received less attention. Therefore, we explored the effects of touchscreen button position and font size on touchscreen operation by older users. The results can be applied to design interfaces improving their quality of life.

## 3 Experimental design

This study explores the effects of variable button positions and font sizes on older users’ operation of the touch screen interface of a popular type of sphygmomanometer, or blood pressure self-monitoring device, this study was approved by the Research Ethics Committee National Tsing Hua University. The device type was chosen for its high market share compared to other medical electronics marketed for home use. This study using factorial design, each subject was asked to operate three buttons (start, measure, and end), and interpreted numbers on the screen. This research tested three hypotheses:

1. There is a difference between younger and older subject groups in the average time needed to complete the same operation on the device.
2. Varying button positions will yield a difference in the average time needed to complete the same operation on the device.
3. Varying font size will yield a difference in the average time needed to complete the same operation on the device.

This study employed an experimental design that utilized current interface controls of common medical electronics marketed for home use. Test subjects were asked to use the sphygmomanometer to monitor their blood pressure, which necessitated the operation of three buttons (start, measurement, and end), according to Karim and Shukur (2016) theory, the most popular font type was Arial; therefore, the buttons were used with Arial text (Karim and Shukur, 2016). The font size is based on Nielsen (2002) theory, it should be minimum 10pt or 12pt if the target group is an older adults group, while Lu (2013) proposes that the text above 22pt can be interpreted clearly, therefore the font size in this study will be between 10-22pt. The dependent and independent variables of the experiment are as follows:

Dependent variables: time for completing the operation of the sphygmomanometer, which involved manipulating the device and interpreting the numbers displayed on the screen.

Independent variables: Font sizes (22-, 18-, 14-, and 10-point) and button position (top, bottom, left, and right).

Button color-matching was based on Ramadan (2011); a white background was combined with a black font. Table 1 shows the CIE coordinates (L, a, and b) and the RGB codes (Table 1).

**Table 1:** CIE and RGB code.

Color	Code					
	CIE (L, a, b)			RGB code value		
	L	a	b	R	G	B
Background						
White	99	0	0	254	254	254
Text						
Black	0	0	0	0	0	0

### 3.1 Subjects

According to Gay (1992), a minimum of 30 subjects were required to allow us to perform comparisons and seek correlations. We included 32 young people aged 18–35 years and 32 older people, aged at least 65 years, thus 64 persons in all, all participants signed an informed consent before participation in our study.

Older subjects were recruited from the Senior Center, while young subjects were recruited from the general college student body. Study recruitment materials informed potential participants of the purpose and experimental process of the study. Criteria for inclusion was as follows: Subjects must: 1) Be able to act independently; 2) Have no major physical or mental injuries; 3) habitually use a computer for 6 months or more prior to study participation; 4) be minimally literate; 5) have adequate vision, or vision corrected to above 0.8 and lacking any major eye condition (e.g., color blindness, amblyopia, or blindness); and 6) be right-handed. Enrolled subjects received a small gift after their participation.

### 3.2 Experimental instruments

**Figure 1:**
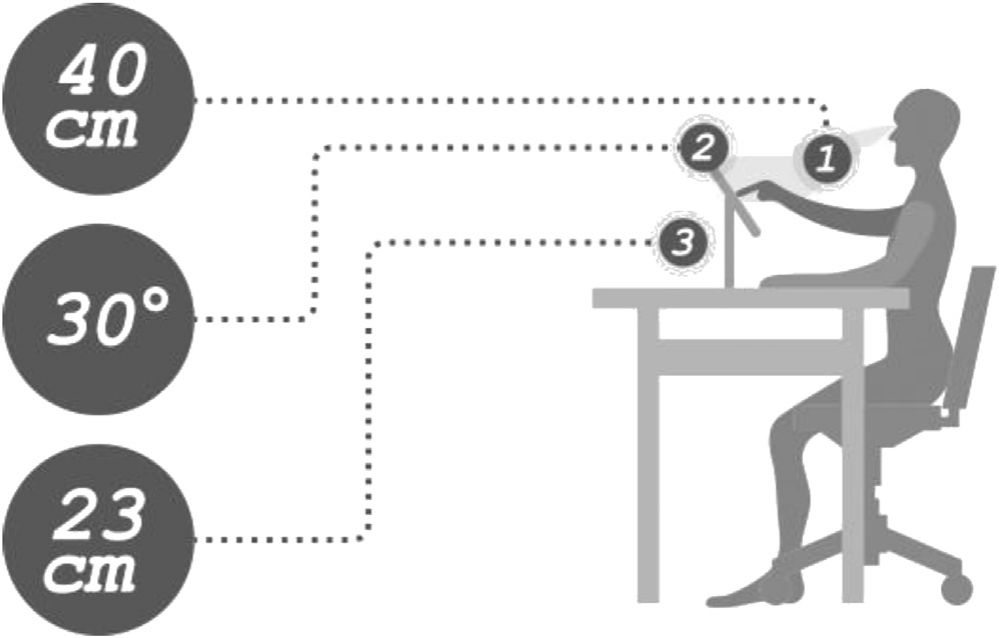
The layout of experimental setting.

This research examined performance while operating a touch-button icon interface. The test environment is based on the theory of Hsu et al. (2017). The experimental stimulus was presented on a 9.7-inch tablet. The computer was placed on a 70-cm-high table with the center of the screen 23 cm distant from the desktop and the screen elevation set to 30°. The test samples on the screen were manipulated one-handedly; researchers recorded all subject reactions. During testing, a support frame was used to hold the sight distance at 40 cm without screen glare (Figure 1).

### 3.3 Experimental environment

To minimize outside interference, we used a classroom at Evergreen College. Sunlight was adequate, noise minimal, and the temperature controlled at 26 °C. Only one subject and one experimenter were present during each test.

### 3.4 Experimental process

Before the formal test, subjects were given a 5-min introduction to the experimental task and its tools, test samples, and operation instructions. Subjects then completed practice exercises to ensure that subjects understood the experimental process and contents.

During the formal test, subjects were asked to visually focus on an "X" in the center of the screen. When the subjects were ready to begin, they could touch the blank space of the screen autonomously, which would trigger the stimulus to appear in the center of the screen. The stimulus screen was composed of three buttons: “start”, “test”, and “end”. The subjects needed to touch the "start" button with one hand, and then the image “000” would appear in the interpretation position. At this time, the three buttons would be rearranged. Then, the subjects would reinterpret and touch the "test" button again. After 3–5 s, a group of numbers would appear on the screen. At this time, the buttons were rearranged again. After the subjects made their interpretation, they would touch the “end” button and then verbally read out the numbers displayed on the screen to complete the first test.

During the experiment, the display position, button size, and color combination were varied randomly. The operation time of the experiment was calculated from the time when the “start” button was activated to the time when the “end” button was activated. Each participant completed 16 tasks (4 button size tests × 4 display position tests), with the total test time lasting approximately 20 min.

### 3.5 Data analysis

Analysis was conducted using the SPSS-PC statistical software package. Descriptive statistics were used to assess each subject's background and mean operation time. Two-way analysis of variance (ANOVA) was used to assess the effects of font size and button position. In the case that the ANOVA showed significance at the p < 0.05 level, the least significant difference (LSD) method was used to conduct a post hoc test.

## 4 Research results

We enrolled 32 young and 32 older subjects, of average ages 31.21 years (range, 18–35 years) and 70.59 years (range, 65–76 years), respectively.

The two groups differed significantly in mean operation time (*F* = 1496.691, *p* = 0.00 < .001); that of the younger group was shorter (M = 2.43, *SD* = 0.72) than that of the older group (M = 5.96, *SD* = 2.83), as revealed by LSD grouping.

The performance of young subjects did not vary significantly by button position (*F* = 1.40, *p* = 0.24˃.05) or font size (*F* = 0.85 *p* = 0.47˃.05), and these variables did not interact (*F* = 0.27, *p* = 0.98˃.05). However, the opposite was true for older subjects (Table 2).

**Table 2:** ANOVA Table for button position, font size.

Source	SS	*df*	*MS*	*F*	*p*	Effect Size
Younger group						
button position	2.22	3	0.74	1.40	0.24	0.004
font size	1.35	3	0.45	0.85	0.47	0.003
button position × font size	1.30	9	0.15	0.27	0.98	0.002
Older group						
button position	42.69	3	14.23	25.03	0.00∗	0.069
font size	7525.26	3	2508.42	4413.21	0.00∗	0.929
button position × font size	64.90	9	7.21	12.69	0.00∗	0.102

∗*p* < 0.01

After LSD grouping, the button display positions fell into four groups, with the top positions associated with shorter operation times, while the left position associated with longer operation times. Font sizes fell into four groups, associated with shorter operation times when 22-point fonts were used were displayed, while longer operation times when 10-point fonts were displayed (Table 3).

**Table 3:** LSD Table of button position, font size for older adults group.

Source	M	*SD*	LSD Group			
button position						
Top	5.65	2.74	A			
Right	5.92	2.72		B		
Bottom	6.07	2.91			C	
Left	6.20	2.94				D
font size (point)						
22	2.21	0.44	A			
18	5.00	0.73		B		
14	7.04	1.02			C	
10	9.60	0.95				D

Table 4 shows the further analysis of interactions between button position and font size revealed that 22-point font, displayed above buttons, was optimally recognized, whereas 10-point font displayed to the left of the buttons was most poorly recognized.

**Table 4:** The table of interactions between button position, font size for older adults group.

Source		M (SD)	SS	*df*	*MS*	*F*	*p*	LSD Group			
top	22	2.03 (0.38)	1690.58	3	563.53	644.77	.000∗	A			
	18	5.14 (0.74)							B		
	14	6.22 (1.32)								C	
	10	9.22 (1.03)									D
bottom	22	2.32 (0.50)	2044.50	3	681.50	1563.86	.000∗	A			
	18	4.65 (0.65)							B		
	14	7.52 (0.58)								C	
	10	9.78 (0.85)									D
left	22	2.30 (0.38)	2087.53	3	695.84	1430.62	.000∗	A			
	18	5.12 (0.78)							B		
	14	7.32 (0.69)								C	
	10	10.07 (0.85)									D
right	22	2.19 (0.44)	1767.55	3	589.18	1234.18	.000∗	A			
	18	5.09 (0.62)							B		
	14	7.10 (0.82)								C	
	10	9.32 (0.82)									D

## 5 Conclusion and suggestions

Physiological changes associated with aging affect daily life. Here, we explored how users operated touch-based interface buttons. From the three hypotheses of this study, we can find out that groups have significant difference in operation times. The results show a striking effect of older group in button position and font size, however, there is no significant difference in younger group. The older group differed from the young in terms of font and button position interpretation. They required more interpretation time, consistent with the results of Goodpaster et al. (2006). How buttons are positioned, and which font size is displayed, lead to significant differences in the interpretation time and operational efficacy among older users. We now discuss the button display position and font size separately.

### 5.1 Button display position

The buttons were displayed on the top, bottom, left, and right. Young subjects found all positions to be equivalent, but differences were found for older users. Top button location maximized operation performance, whereas buttons on the left were associated with the poorest performance. The interface buttons of traditional medical products (such as sphygmomanometers) are mostly on the bottom and the right. Such placement may affect touch interface performance, but more research is needed.

### 5.2 Button font size

Font size did not affect task performance by young subjects (who completed tasks within 2.43 s) but it did significantly affect the performance of the older subjects. Older users take longer to complete the same operation on devices, consistent with literature findings (Ziefle, 2010**)**. When 22-point font was displayed, however the operation time of older subjects (M = 2.21, *SD* = 0.44) was similar to that of younger subjects.

The physiological changes associated with aging are often associated with lower quality of life. Although older populations use many technological products, the ubiquity of touch-based interfaces means that they will be increasingly used by older people who need to use these technologies to self-monitor their health. For older users, identification errors or false touches are the most common issues that compromise effective operation. Therefore, greater understanding of the abilities and physical limitations of older populations is needed. While this study focused on operation at the word level, or presentation position of interface buttons, future research may explore interface button icons and colors that may be more effective for older users. The results can be widely used in touch interface products, such as: sphygmomanometers, interphones, measuring instruments, etc. It is worth noting that the age-old society leads to an increasing number of older adults living alone, so the probability of contacting with family/nursing staff through mobile phones/telecare is going to increase in the future; therefore, it is understood that the interface design or related products will undoubtedly become a trend. We hope that the conclusions of this study will inform interface researchers, designers, and care workers involved with older populations about future research and design, which may lead to the design of appropriate healthcare products for older populations.

## Declarations

### Author contribution statement

PO-CHAN YEH: Conceived and designed the experiments; Performed the experiments; Analyzed and interpreted the data; Contributed reagents, materials, analysis tools or data; Wrote the paper.

### Funding statement

This work was supported by 10.13039/501100004663Ministry of Science and Technology (MOST 105-2410-H-364-008).

### Competing interest statement

The authors declare no conflict of interest.

### Additional information

No additional information is available for this paper.
